# Touch DNA: impact of handling time on touch deposit and evaluation of different recovery techniques: An experimental study

**DOI:** 10.1038/s41598-019-46051-9

**Published:** 2019-07-02

**Authors:** Francesco Sessa, Monica Salerno, Giuseppe Bertozzi, Giovanni Messina, Pietrantonio Ricci, Caterina Ledda, Venerando Rapisarda, Santina Cantatore, Emanuela Turillazzi, Cristoforo Pomara

**Affiliations:** 1 Department of Clinical and Experimental Medicine, Section of Legal Medicine, University of Foggia, Foggia, Italy; 2 Department of Clinical and Experimental Medicine, University of Foggia, Foggia, Italy; 3 Institute of Legal Medicine, Università degli Studi Magna Graecia di Catanzaro, Catanzaro, Italy; 4 Department “G.F. Ingrassia” - Section of Hygiene and Public Health, University of Catania, Catania, Italy; 5 Department of Surgical, University of Pisa, Pisa, Italy; 6 Department of Medical and Surgical Sciences and Advanced Technologies GF Ingrassia, University of Catania, Catania, Italy

**Keywords:** PCR-based techniques, DNA sequencing

## Abstract

“Touch DNA” is DNA obtained from biological material transferred from a donor to an object or a person during physical contact. This particular kind of evidence could play an essential role in forensic laboratory work and is considered an important tool for investigators. Even though the principal aspects of “Touch DNA” have been extensively studied, to date, there are few reports in the research field of DNA retrieval from garments that have been worn. This study aimed to investigate the “handling time”, analyzing particularly the minimum contact time required to deposit a sufficient amount of DNA on a garment to produce an interpretable profile of the “handler”. Moreover, three different sampling techniques were compared (“dry swab”, “cutting out”, and “adhesive tape”) with the aim of defining the technique that guarantees the best recovery of the three methods tested. Analyzing the data of this experimental model, a “handling time” of two seconds is enough to release sufficient DNA on to a garment to obtain a complete profile. Moreover, this study demonstrated that when targeting for foreign DNA, the sample area should be narrowed down as much as possible to the smallest area possible to maximize target DNA recovery.

## Introduction

“Touch DNA” is DNA obtained from shed skin cells and other biological material transferred from a donor to an object or a person during physical contact^1,2^. This particular kind of evidence could play an essential role in forensic laboratory work and is considered an important tool for investigators^3^. “Touch” DNA refers to the collection of minute biological samples at the crime scene or extracting tiny amounts of material from a sample in a forensic laboratory. Based on Locard’s Exchange Principle, which states, “every contact leaves a trace”^4^, the collection of “touch” DNA, to obtain significant profiles from different surfaces, remains an important procedure in forensic investigations.

Genetic profiles generated from fingermarks were first described in 1997 by RAH van Oorschot *et al*.^5^. Fingermarks refer to the marks left by the papillary ridge patterns present on fingers, palms, toes, and soles on touched surfaces^6^. Fingermarks are essential forensic evidence used in a wide range of forensic investigations helping to generate a DNA profile for human identification^5^. This kind of evidence is very useful in a wide range of criminal investigations ranging from theft, sexual violence, to murder. For instance, very important evidence could be collected by analyzing the steering wheel of a vehicle used in a theft, or weapons, and clothes in cases of murder or sexual assault. Moreover, the collection of “Touch DNA” with the aim of identifying a person of interest from a crime scene could be very useful especially in the absence of body fluids^7–9^.

Even though the principal aspects of “Touch DNA” have been extensively studied^10–13^, to date, there are few reports in the research field of DNA retrieval from garments that have been worn^14–21^. In criminal cases, sampling techniques are very important to collect the best evidence. One of the most common methods for optimal collection of cellular material is the so-called “swab technique”, using sterile cotton swabs on the surface of the object^22^. To improve the quality of the resulting DNA profiles, the double swab technique (wet and dry) is usually applied^23^. Another sampling technique frequently used in a large number of forensic laboratories is “cutting out” the area of interest; this method is applied especially to soft items^24,25^. Moreover, the “adhesive tape” lifting technique has been used for years for DNA profiling^26^. This last sampling method is quick and straightforward but the DNA extraction is challenging due to the stickiness, rigidity, and size of the tape^27^.

This study aimed to investigate the “handling time”, analyzing particularly the contact time needed to deposit a sufficient amount of DNA on a garment to produce an interpretable profile of the “handler”. Moreover, three different sampling techniques were compared (“dry swab”, “cutting out”, and “adhesive tape”) with the aim of defining the technique that guarantees the best recovery of the three methods tested.

## Methods

### Substrates and contact simulations

Ten female volunteers (“wearers”) wore a new brassiere (previously irradiated with UV rays for 20 min to remove exogenous DNA) for more than 12 h (from 8 pm to 8/9 am, minimum 12 h and maximum 13 h), under normal conditions (during this time they carried out their usual activities such as walking about, eating, sleeping). Prior to the experiment, the lateral regions of the brassieres were marked in 8 areas (4 for each side).

Each brassiere was then placed in a sterile “acid-free” plastic bag. Subsequently, after 1 h, the brassieres were removed from the sterile bag by 10 male volunteers (“handlers”), each assigned to a specific brassiere. Moreover, they held each region of the brassiere for different time intervals with the same two fingers (thumb and index) for each hand. Particularly, the two fingers moved on two opposed specific region of the brasserie for the established time, depositing DNA on the brassiere. Prior to this, they had washed their hands 2 h before the experiment, to allow for sufficient DNA transfer. Furthermore, after the first touch and between touches, they were invited to avoid contact with other subjects, handling only their own personal objects. Moreover, between each application, an interval of at least 60 min was maintained.

For each handler a set of eight different handling times on brassieres was recorded: 60 s for region A, 45 s for B, 30 s for C, 20 s for D, 10 s for E, 8 s for F, 5 s for G, and 2 s for H. Lateral regions were chosen because they are in full contact with the “wearer’s” skin. Reference profiles of both handler and wearer were obtained with buccal swabs.

**Figure 1:**
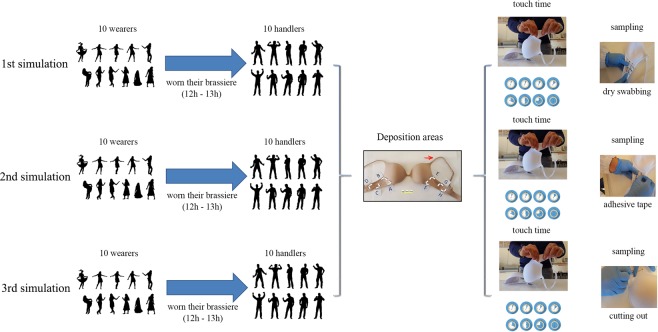
The experimental model is summarized in this picture. Three simulations were performed, one for each sampling method. Moreover, for each simulation 8 DNA depositions (4 for each side) with different contact times were carried out. At the end of the simulations, 240 samples were collected.

For consistency, and for the purpose of this study, the same model of brassieres was used with bright textiles (white and beige) made of cotton (85%) and elastane (15%). The experimental model is illustrated in Fig. 1.

All procedures were approved by the Scientific Committee of University of Foggia (Italy).

All participants gave their written informed consent to take part in the study and signed permission for obtaining DNA profiles and all procedures were performed in accordance with the Declaration of Helsinki. The participants were free to withdraw their participation at any time during the course of the study.

### Sampling

Each experiment was carried out in triplicate using the same couple “handler-wearer”. This condition was applied with the aim of detecting the expected DNA profiles (handler and/or wearer), even if it cannot be ruled out that some extraneous DNA may be picked up by the participants while performing their everyday tasks. Negative controls - unworn brasseries of the same material and brand after irradiation with UV rays and before the experimentation phases - were investigated, obtaining 30 control samples. To collect DNA from the brassieres three recovery methods were used, deposit area ≅1.5 cm^2^: 1- sampling with dry-swab (Copan, Brescia, Italy) that was rubbed with moderate pressure and rotation on both surfaces (internal and external). The use of a dry-swab was chosen for this experimental model because it was used on a porous surface; in this manner, a possible contamination of the other deposition areas was avoided (which could have happened using the wet/dry double swabbing method); 2- sampling the selected area by cutting it, using sterile scissors, into small pieces; 3- sampling the internal and external area with adhesive tape (Sirchie® Fingerprint Laboratories, Youngsville, NC, USA) as previously described^26,28^. A total of 240 samples were obtained.

### DNA analysis

All samples were placed in a 1.5 mL tube and DNA extraction was performed following the QIAmp® DNA Investigator Kit protocol (QIAGEN Vic, AUS), with minor modifications (overnight incubation at 56 °C, mixing the samples several times.). DNA was then eluted into 30 μL of AE buffer. The concentration of all DNA extracted was determined with the use of the Quantifiler Duo DNA Quantification kit (Applied Biosystems, Europe BV), according to the manufacturer’s guidelines. Duplicate Quantifiler standards ranging from 50 ng/μL to 0.023 ng/μL and duplicate negative controls were processed in tandem with the reactions. Both reactions were carried out on the ABI PRISM 7500 Sequence Detection System (Applied Biosystems, Europe BV).

All samples were amplified with the Identifiler Plus Amplification Kit (Applied Biosystems): input trace DNA volume was set to 10, 5, 1 μL at a concentration range of 0–0.062, 0.0625–0.125, 0.500–1.000 ng/μL, respectively, in 25 μL PCR final volume. Samples of higher concentrations were diluted down to 1 ng/μL. Amplification was performed by PCR on an Applied Biosystems (Forest City, CA, USA) 9700 thermal cycler following the manufacturer’s specifications.

A positive control, using the 9947 A DNA template, and a negative control, to monitor amplification success and reagent contamination, were also amplified. The amplified product was analyzed in a 10 μL reaction that consisted of 1 μL amplified product plus 9 μL formamide/GeneScan^TM^ 500 LIZ^TM^ dye size standard (Applied Biosystem) mixture, using capillary electrophoresis on an AB 3130 (Applied Biosystem) instrument with Gene-Mapper.

The DNA profiles obtained were analyzed and labeled as unknown profiles. They were subsequently analyzed using an analytical threshold of 50 RFU. In the mixture interpretation, the criteria used to define major or minor contributors were based on peak proportions and peak height ratios (PHR). Considering that PHRs become more varied and tend to have a lower value as the amount of input DNA decreases, a single PHR expectation was applied (60%).

### Statistical analysis

Descriptive analyses were performed using frequency percentages (total number of alleles observed/total number of alleles expected). The alleles shared among the handler and wearer at any of the loci were counted for both profiles. The “drop in” alleles were not considered for analysis (no more than 1–2 alleles were observed). Data were analyzed with the software SPSS 22.0 for Windows. The one-way variance analysis (ANOVA) was used to determine any statistically significant differences among the groups. This test was applied to the ratio (alleles observed/alleles expected) from each subject, standardizing the different number of alleles.

## Results

In this study, DNA profiles of “handlers” were frequently found as the major contributor and these findings were consistent with all three methods of sampling: “adhesive tape” (AT), “dry swabbing” (DS) and “cutting out” (CO).

**Figure 2:**
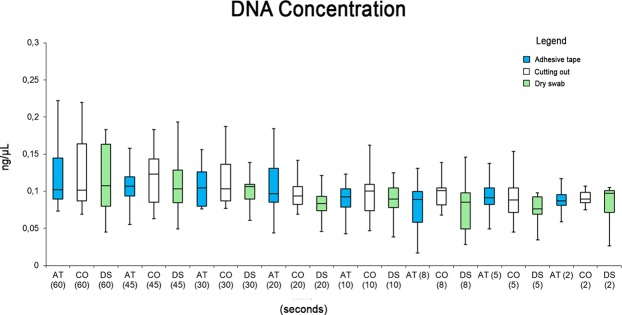
Box plot summarizing the DNA concentrations for all extraction techniques and for handler time.

The values of DNA concentration are summarized in Fig. 2. No statistical differences were reported in the box plot analysis considering both the handler time and the extraction techniques (one-way ANOVA [F(2,21) = 3.46, p = 0.52]).

The “Wearer” DNA profiles were found as the major contributor in the mixture in only 5 cases: these 5 tests were sampled with “adhesive tape” (Table 1).

**Table 1:** Distribution of profiles in relationship to the techniques used and profiles obtained.

Relationship Between Technique and Profile Obtained					
	“Handler” incomplete profile	“Handler” complete profile	“Handler” major and “wearer” incomplete profile	“Handler” major and “wearer” minor profile	“Wearer” major and “handler” minor profile
AT	7	20	46	2	5
CO	1	30	49	0	0
DS	7	28	45	0	0
TOTAL *(Male:Female DNA Ratio - Mean)*	**15**	**78**	**140** (1:0.12 ± 0.06)	**2** (1:0.43 ± 0.08)	**5** (1:1.18 ± 0.09)

Legend: AT = Adhesive Tape; CO = Cutting Out; DS = Dry Swab; incomplete = profile with more than two drop-out alleles; complete profile = full profile or with maximum two drop-out alleles.

The data are summarized as a percentage (number of alleles observed/number of alleles expected, averages across people and repeats), and are reported in Table 2. The Amelogenin locus was excluded from this statistical analysis.

**Table 2:** Distribution of profiles as a percentage (number of alleles observed/number of alleles expected, averages across people and repeats and standard deviation).

Percentage of Profiles (*averages across people and repeats*)																
	60 s		45 s		30 s		20 s		10 s		8 s		5 s		2 s	
	H	W	H	W	H	W	H	W	H	W	H	W	H	W	H	W
AT	87.5 ± 0.37	38.3 ± 0.36	98.1 ± 0.75	33.5 ± 0.36	97.7 ± 0.75	50 ± 0.72	98.4 ± 0.75	36.1 ± 0.73	94.3 ± 0.37	28.8 ± 0.36	96.2 ± 0.37	32.4 ± 0.36	90.2 ± 0.37	24.8 ± 0.36	89.0 ± 0.75	43.0 ± 0.36
CO	98.8 ± 0.37	16.7 ± 0.36	99.2 ± 0.37	18.6 ± 0.36	99.2 ± 0.37	32.8 ± 0.36	98.4 ± 0.37	32.4 ± 0.36	98.4 ± 0.75	29.5 ± 0.36	98.4 ± 0.75	13.1 ± 0.36	92.1 ± 0.37	27.7 ± 0.36	89.4 ± 0.37	46.3 ± 0.36
DS	92.1 ± 0.75	13.1 ± 0.72	93.9 ± 0.37	16.0 ± 0.36	95.1 ± 0.37	25.5 ± 0.36	94.3 ± 0.75	25.1 ± 0.36	95.8 ± 0.75	16.0 ± 0.36	96.2 ± 0.75	22.6 ± 0.36	93.6 ± 0.37	25.5 ± 0.36	94.7 ± 0.37	35.7 ± 0.36

Legend: AT = Adhesive Tape; CO = Cutting Out; DS = Dry Swab; H = Handler; W: Wearer.

**Figure 3:**
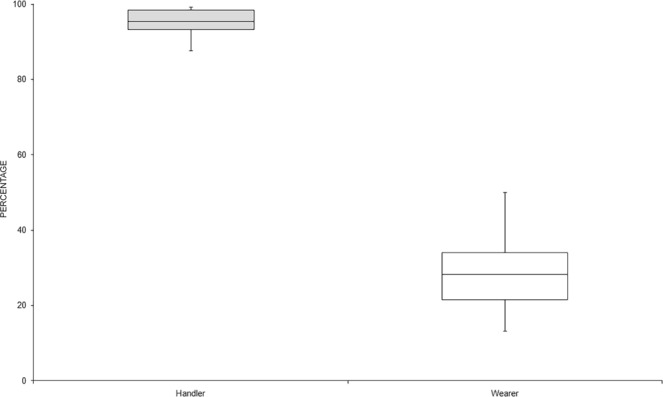
Box plot summarizing the recovered profiles (data expressed in percentage).

Moreover, the data are schematized in a box plot, considering the percentage of profile recovered (Fig. 3). There were statistically significant differences among group means as determined by one-way ANOVA [F(1.46) = 920.85, p = <0.05 (4,55 × 10^−32^)]: the handler profiles were recovered in significantly higher number with respect to the wearer profiles.

**Figure 4:**
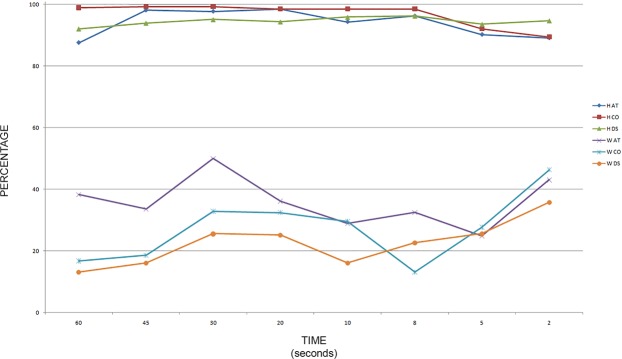
Graph summarizing the recovery percentage (alleles observed/expected) for “adhesive tape” (AT), “cutting out” (CO), and “dry swabbing” (DS), considering the collected profiles “handler” (H) or “wearer” (W) in relation to the handling time.

The results obtained were analyzed in relationship to the sampling techniques and to the handling time (Fig. 4).

**Figure 5:**
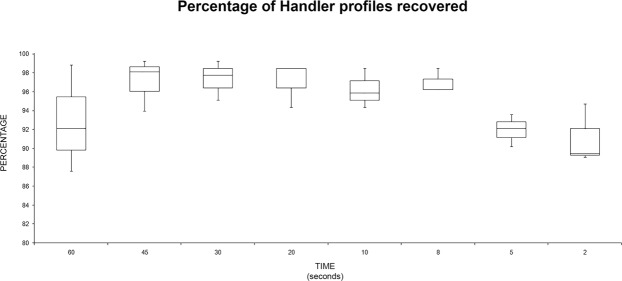
Box plot summarizing the recovery percentage (alleles observed/expected) for “handler” profiles recovered related to the handling time.

As summarized in Fig. 5, the percentage of recovered “handler” profiles decreased at short times (5 and 2 s); moreover, at 60 s there was a great variability in the recovered profiles. This evaluation was obtained without considering the recovery techniques but only the handling time. No statistically significant differences were found among group means with one-way ANOVA [F(7,16) = 2.43, p = 0.066].

**Figure 6:**
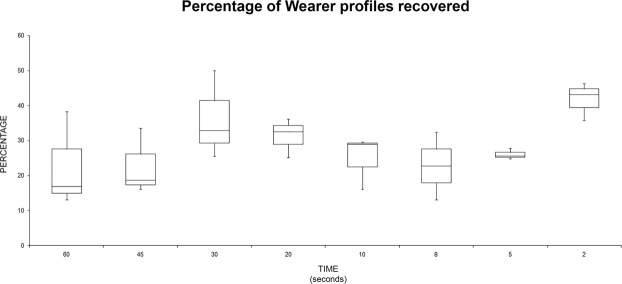
Box plot summarizing the recovery percentage (alleles observed/expected) for “wearer” profiles recovered related to the handling time.

The same analysis was performed with the “wearer” data (Fig. 6): the best recovery for the “wearer” profiles was recorded at the time of two seconds. No statistically significant differences were found among group means with one-way ANOVA [F(7,16) = 1.91, p = 0.134].

Finally, the data were summarized as percentage, in order to analyze the variation of the sensitivity of each sampling method, observing the “handler” and the “wearer” profiles.

**Figure 7:**
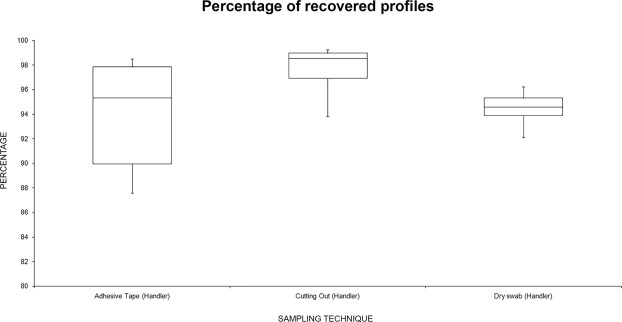
Box plot summarizing the recovery percentage (alleles observed/expected) for “handler” profiles recovered related to the sampling technique.

Figure 7 shows that the best values for “handler” profiles were obtained with the “cutting out” technique. However, it is important to note that no statistically significant differences were found among techniques with one-way ANOVA [F(2,21) = 1.52, p = 0.24].

**Figure 8:**
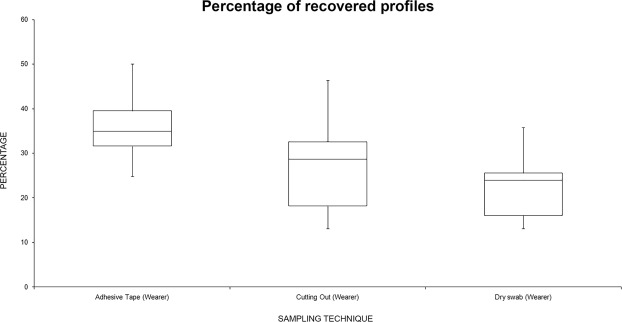
Box plot summarizing the recovery percentage (alleles observed/expected) for “wearer” profiles recovered related to the sampling technique.

Figure 8 summarizes the data analyzing the recovery percentage for “wearer” profiles related to the sampling technique: “adhesive tape” was the best technique for “wearer” sampling. There were statistically significant differences among group means as determined by one-way ANOVA [F(2,21) = 4.77, p = 0.019]. Moreover, comparing, singularly, this technique with the others, it was significantly better compared with “dry swabbing” [F (1,14) = 12.3, p = 0.003], while it was not significant when compared with “cutting out” [F (1,14) = 3.37, p = 0.087]. There were no significant differences in the recovering of wearer profiles comparing “cutting out” vs “dry swabbing” [F(1,14) = 4.6, p = 0.32].

All negative controls including the unworn brasseries showed no profiles. Finally, 13 profiles showed a “drop in” DNA (1–2 alleles per profile): this result could be linked with the secondary or indirect DNA transfer during the daily activities of the wearer or the handler.

## Discussion

It is a known fact in the forensic community that the recovery of DNA profiles is related to many factors when touch DNA is collected from the clothing of a victim or suspect. Unmanageable factors, such as the type of contact, shedder status of victim and offender, and environmental conditions, can play significant roles in touch DNA recovery. In this study, 10 “handler” males were invited to touch the brassieres worn by 10 “wearer” females for different times: this experimental model was used to enhance the inter-individual variability, considering the presence of good or bad shedders^28,29^. The first step in collecting touch DNA is to target the relevant area. Touch DNA on clothing is normally not visible even under a forensic polilight source. Usually, the sampling should be restricted to the area of interest, avoiding the error of sampling the wrong area and thus obtaining a DNA profile not useful for the investigation, with the presence of unknown or “drop in” alleles that could be related to secondary/indirect DNA transfer. On the other hand, the selection of the appropriate recovery method should be standardized to obtain an optimal DNA profile.

In this study, three sampling techniques for detecting touch DNA were compared: “adhesive tape”, “cutting out” the area of interest and “dry swab”. The data reported here show that in recovering the “handler” there were no statistically significant differences among the three techniques analyzed, even if the “cutting out” technique showed the greatest recovery. However, analyzing the data of the “wearer”, the sampling with “adhesive tape” showed the best result among the three techniques: this result was statistically significant. “Dry swab” sampling seems the worst technique for DNA profiling in the proposed experimental model, even though at the shortest periods of contact, this technique yielded the highest number of “handler” profiles recovered compared to the other techniques.

The reported data show that “adhesive tape” and “cutting out” of the area are the two most efficient and sensitive techniques. Nonetheless, each method has its own limitations: the adhesive tape technique is quick and straightforward, however, DNA extraction may require some adjustment, according to Forsberg *et al*.^19^. On the other hand, “cutting out” the area of interest does not conserve the garment; moreover, it cannot always be applied because not every surface can be cut out. Another problem related to “cutting out” is that both inner and outer surfaces are sampled at the same time increasing the chance of wearer DNA swamping any traces of foreign DNA present and/or generation of uninterpretable complex mixtures.

The low DNA recovery observed with the “dry swabbing” technique could be related to the use of a single swab without a moistening agent, which is required to dissolve epithelial cells from the substrate. Indeed, in other study^30^ and in the forensic laboratories, a wet and dry swabbing technique is often used, as it has been accepted as an optimal method for DNA collection when using swabs. Although the use of this sampling technique is undoubtedly a limitation of this experimental model, the dry-swab was applied in this particular experimental design with the aim of avoiding contamination in the contiguous deposition areas, considering the porous surface.

The effects of the handling time were subsequently analyzed to determine if it had any impact on detection of “handler” and/or “wearer” profile. Complete “handler” profiles were found with a high percentage (87.6% to 99.24%) at different touch times, with all sampling methods; the “handler” was the major contributor compared to the “wearer” in all mixed DNA profiles, with the exception of five tests.

The handler was detected in all 240 samples analyzed. In all DNA mixtures, the “wearer” DNA profile was detected when the total DNA concentration was lower than 70 pg. However, in the samples with higher DNA concentrations, we frequently detected only the “handler” profile: it was indicative that the “handler” profile could “overwrite” the “wearer” profile.

The discussed data seem in accordance with Meakin and Jamieson’s review, nevertheless, the friction was not tested^31^. In particular, even if it is commonly thought that the amount of DNA deposited on a surface could be increased with increased handling time, the reviewed data suggest the length of contact is not a significant factor.

## Conclusion

This experimental work was performed in particular settings. In fact, the principal limitation of this study design and thus the limitation of the generated results, is related to the sampling area. In routine forensic casework, when sampling for wearer DNA, the target area is usually on the inside and larger than the surface sampled in this experimental model: for examples, Petricevic *et al*. sampled approximately 3 cm^2^ of fabric in their study^32^, while Dong *et al*. sampled about 4 cm^2^ ^24^. The entire area of such a sample is likely to contain the DNA of the wearer, while the DNA of the handler is likely to be confined to a small area where the garment was touched. For these reasons, in other papers and in real casework, the wearer was frequently detected as a single or a major component^18,20,21^.

Nevertheless, this study provides insights into the effect of the duration of deposition of touch DNA and on the techniques used to recover it. As previously described, in another experimental study a handling time of 15 s was successful in releasing enough DNA, deemed useful for detecting the “handler” as the major contributor^10^. In this experimental model, the results show that the person handling the garment last contributes the most even though he/she may touch the garment for merely a few seconds (even only 2 seconds); these findings are not influenced by the couple “handler/wearer”.

The discovery of “touch DNA” has broadened the investigators’ horizon since it is possible to obtain genetic profiles from a crime scene in the absence of biological fluids. On the other hand, uncertainties regarding “touch DNA” remain, as “touch DNA” often fails to provide a straightforward and precise answer to the judge. Taking into consideration that clothes are often used as evidence from a crime scene, this study demonstrates that it could be possible to obtain a DNA profile that belongs to the person who had simply just touched the garment. For these reasons, a new insight into forensic genetics is related to the application of mRNA/miRNA technologies to identify the source origin. In the last years, significant researches into developing more definitive, molecular-based, methods for the conclusive identification of all forensically relevant body fluids were performed^33–35^. Moreover, in order to identify the anatomical region of the skin cells sampled, Lindenbergh *et al*.^36^ described a different expression of the mRNAs set used in their study linked to different areas of skin.

More knowledge on the frequency of detection of wearer and/or handler DNA profiles will allow scientists to evaluate the likelihood of obtaining a matching profile if an individual wore a garment rather than simply touching the garment in disputed case scenarios.

Overall, this study demonstrates that the presence of a single DNA profile or the major contributor to a mixture obtained sampling a worn garment may not necessarily be the wearer. Moreover, it should also affect the common practice of generating profiles for a missing person using their clothes, and warrants the need for checking a number of garments instead of just a single garment. This procedure should eventually be applied when there are no toothbrushes, hairbrushes and/or samples from relatives. In this regard, the results of the present study highlight the importance of the sampling area, related to the aim of forensic investigation. Indeed, in casework, when targeting clothing for wearer DNA (as opposed to foreign DNA) only the inner surface is sampled, specifically to avoid foreign DNA that is known to be present mostly on outer surfaces. On the other hand, these experimental data showed that when targeting for foreign DNA, the sample area should be narrowed down as much as possible to the smallest area possible to maximize target DNA recovery. Moreover, the contact duration does not appear to have an effect on the amount of DNA deposited and when a major contributor is detected on a garment, based on this experimental study, it provides an indication of direct contact as opposed to indirect transfer.

Finally, this study highlights an important aspect: everyone in the medico-legal community - forensic scientists and technicians, DNA analysts, potential jurors, judges and lawyers for both the prosecution and defense - must know the power of touch DNA and understand the potential limitations of this technique.
